# Get Over It! A Multilevel Threshold Autoregressive Model for State-Dependent Affect Regulation

**DOI:** 10.1007/s11336-014-9417-x

**Published:** 2014-08-05

**Authors:** Silvia De Haan-Rietdijk, John M. Gottman, Cindy S. Bergeman, Ellen L. Hamaker

**Affiliations:** 1 Methodology and Statistics, Faculty of Social and Behavioural Sciences, Utrecht University, P.O. Box 80140, 3508 TC Utrecht, The Netherlands; 2 Relationship Research Institute, Seattle, WA USA; 3 Department of Psychology, University of Notre Dame, Notre Dame, IN USA

**Keywords:** dynamical modeling, intensive longitudinal data, threshold autoregression, bayesian estimation, affect regulation

## Abstract

Intensive longitudinal data provide rich information, which is best captured when specialized models are used in the analysis. One of these models is the multilevel autoregressive model, which psychologists have applied successfully to study affect regulation as well as alcohol use. A limitation of this model is that the autoregressive parameter is treated as a fixed, trait-like property of a person. We argue that the autoregressive parameter may be state-dependent, for example, if the strength of affect regulation depends on the intensity of affect experienced. To allow such intra-individual variation, we propose a multilevel threshold autoregressive model. Using simulations, we show that this model can be used to detect state-dependent regulation with adequate power and Type I error. The potential of the new modeling approach is illustrated with two empirical applications that extend the basic model to address additional substantive research questions.

**Electronic supplementary material:**

The online version of this article (doi:10.1007/s11336-014-9417-x) contains supplementary material, which is available to authorized users.

Human behavior is a complex product of interrelated factors, such as cognition, emotion, psychophysiological factors, and social interactions, all of which vary over time. For this reason, longitudinal studies are important, because they can provide insight into the dynamics of a psychological process as it unfolds over time. This is especially true for longitudinal research designs that include many measurements, resulting in time series data, also referred to as intensive longitudinal data (Walls & Schafer, 2006). While intensive longitudinal research designs have gained popularity, partly due to technological developments, statistical tools for the analysis of time series data can be further developed and refined to improve their potential, especially for the analysis of samples of multiple persons. One type of time series model that has found applications in psychology, is the autoregressive (AR) model. Multilevel extensions of this model have proven useful in studies of regulatory processes, such as affect regulation (Suls, Green, & Hillis, 1998; Kuppens, Allen, & Sheeber, 2010) and alcohol use (Rovine & Walls, 2006). However, they are limited when it comes to studying intra-individual variation.

In this study, we combine the advantages of several available models to develop a new extension that allows more flexible modeling of regulation in intensive longitudinal data. More specifically, building on the threshold-autoregressive (TAR) model for a single time series (Tong & Lim, 1980), we develop a multilevel TAR model that can be used to investigate more diverse hypotheses about affect regulation. Compared with the multilevel AR model, the proposed model can be used similarly to study interpersonal differences in regulatory processes, but it also allows researchers to study intra-individual variation more closely: where the AR model assumes that each person’s regulation is characterized by a fixed trait-like property, the TAR model allows regulation to be state-dependent within the individual.

In this paper, we focus on affect regulation, but note that these models could also be used for the study of other regulatory processes. This paper is organized as follows. First, we discuss the substantive background of affect regulation studies, that led us to the current model extension. Next, we describe the existing AR and TAR models and we present the basic multilevel TAR model for state-dependent regulation. We then consider the feasibility of distinguishing between trait-like and state-like regulation by discussing the results of a simulation study. We used simulated data sets with varying numbers of persons and measurements to evaluate the power and Type I error rate for testing whether regulation is state-dependent, and we also consider the accuracy of the multilevel TAR model estimates. In the following section, we present two empirical applications that extend the basic model: In the first one, we study observational data concerning the affective behavior of spouses during a conflict discussion, and in the second one, we analyze diary data concerning negative affect. We conclude the paper with a discussion of limitations of the proposed modeling approach, as well as several other ways that the multilevel TAR model could be extended to address different types of research questions.

## Background

To model the dynamics of affect regulation, and to study individual differences therein, Suls et al. (1998) analyzed time series data from multiple individuals using a multilevel AR model. In that model, each observation is regressed linearly on the previous observation, and the regression coefficient is a random effect (i.e., it varies between persons). When a person’s autoregressive coefficient is high, this means that it takes them relatively long to “recover” after an external event causes a change in affect. In other words, persons with a higher autoregressive coefficient have more spill-over of affect into consecutive observations. For this reason, the autoregressive coefficient in the multilevel AR model is also called the *inertia*, and has been interpreted as a measure of *regulatory weakness*.

Suls et al. (1998) found a positive relationship between neuroticism and inertia, obtained with the multilevel AR model. In another study by Kuppens et al. (2010), using the same modeling approach, inertia was also found to be positively related to depression and low self-esteem. Since neuroticism, depression and low self-esteem are associated with many psychological disorders, these findings may indicate that weak affect regulation, as characterized by high inertia, is involved in psychological maladjustment, in general. For this reason, we argue that it is important to conduct further studies investigating the dynamics of affect regulation and its relationship with other person characteristics.

In the above studies of Suls et al. (1998) and Kuppens et al. (2010), inertia was modeled as a time-invariant characteristic of a person, in the sense that there was a single inertia parameter per person. This is a strong assumption, and it seems more realistic that regulatory weakness is actually state-dependent, meaning that it varies within a person over time. Support for this idea comes from Koval and Kuppens (2011), who found that they could experimentally manipulate a person’s inertia. However, the AR modeling approach is limited and cannot be used to study state-dependent inertia when the states are not clearly separated in time or by experimental conditions.

After considering the findings above, we propose finding out whether inertia may differ between episodes of increased and decreased affect, but this cannot be studied using the multilevel AR model. More specifically, we hypothesize that depressed or neurotic persons may have weaker affect regulation *only or especially* during episodes of increased negative affect, because they are more likely to start ruminating during episodes that are characterized by high-intensity negative affect. Rumination is the tendency to keep reflecting on negative experiences or thoughts, and it has been shown to inhibit problem solving (Lyubomirsky & Nolen-Hoeksema, 1995; Lyubomirsky, Tucker, Caldwell, & Berg, 1999), and to exacerbate or prolong negative affect (Lyubomirsky & Nolen-Hoeksema, 1993; Donaldson & Lam, 2004; for an extensive overview concerning rumination, see Nolen-Hoeksema, Wisco, & Lyubomirsky, 2008). Furthermore, in a related vein, Pyszczynski and Greenberg (1987) describe what they refer to as the *depressive self-focusing style* of individuals whose self-focus is much greater when negative events are salient, than when positive events are.

The multilevel TAR model that we develop in this study is suitable for testing this hypothesis, and can be used in a broader context for investigating various proposed mechanisms of state-dependent regulation. Because the model contains multiple inertia parameters per person, it does not assume that regulatory weakness is a trait-like property, but allows more flexible modeling of intra-individual variation in addition to interpersonal differences in regulatory strength.

## AR and TAR Models for Affect Regulation

We begin with discussing the AR(1) model, in which there is a single inertia parameter for a person, and its multilevel extension. Next, we discuss the TAR(2,1,1) model (Tong & Lim, 1980), in which a person has two inertia parameters, so that affect regulation can be state-dependent. In addition, we present our multilevel extension of the TAR model, which can be used to investigate interpersonal differences in state-dependent affect regulation.

### The AR Model for a Single Person

**Fig. 1:**
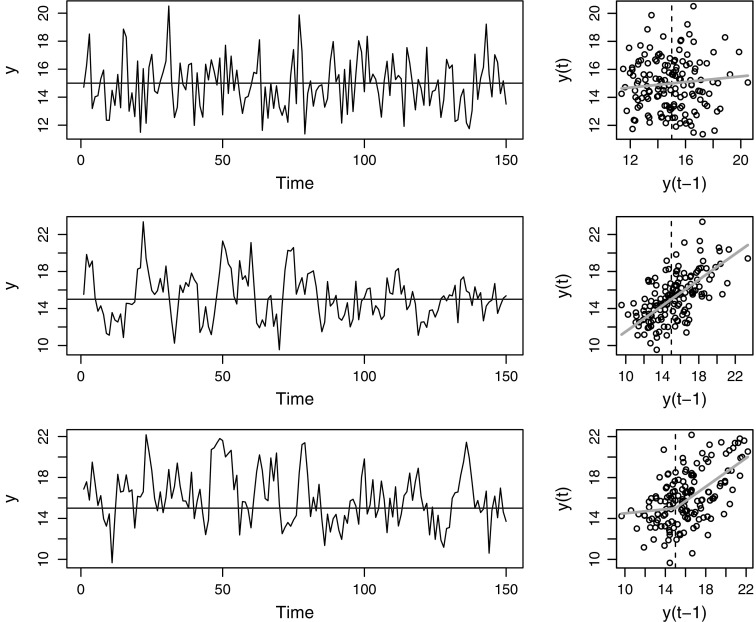
Hypothetical negative affect scores for persons A, B and C, and corresponding state-space plots depicting the underlying autoregression. All three persons have the same equilibrium (15). Persons A and B are described by AR models with inertias ($\phi$) of 0.1 and 0.7, respectively. Therefore, person A is quicker to recover toward his equilibrium, and person B is characterized by more carry-over affect from one moment to the next, indicating regulatory weakness. Person C is described by a TAR model with $\phi = 0.7$ during episodes of increased negative affect ($>$15), and $\phi = 0.1$ during decreased negative affect ($<$15). Thus, person C has weaker affect regulation during episodes of increased negative affect.

In the AR(1) model for affect regulation, there is one inertia parameter reflecting a person’s regulatory weakness, and this is the regression coefficient that links each observation to the immediately preceding observation. To illustrate this model, which we will simply call the AR model hereafter, we consider an example of two hypothetical persons. The time series plots in the upper two panels of Figure 1 depict their negative affect scores on 150 consecutive measurements, with higher scores indicating more intense negative affect. The horizontal line represents the *equilibrium* of the person, the baseline level of negative affect that the person tends toward. The time series plots show that the negative affect of both persons fluctuates around their equilibrium over time and that there is no long-term trend. The difference between the two persons is in their autoregressive coefficient $\phi$, which represents their emotional inertia for negative affect. Person A has a $\phi$ of 0.1, while that of person B is 0.7. This implies that person B is characterized by more regulatory weakness, causing a larger carry-over of negative affect from one occasion to the next. It can be seen in Figure 1 that person B is more likely to have several consecutive scores above or below his/her equilibrium, while person A’s affect is quicker to recover toward his/her equilibrium.

Another way of looking at these data is by plotting each observation against the previous observation, as depicted in the state-space plots on the right side of Figure 1, where the diagonal line depicts the autoregressive relation (based on the $\phi$ parameter) underlying the scores. It can be seen that the regression line, regressing the current affect score on the previous one, has a steeper slope for person B than for person A, and this is because the autoregressive coefficient $\phi$ is higher for person B.

The AR model underlying the data for persons A and B is $y_{t} = \alpha + \phi y_{t-1} + \epsilon _{t}$ , where $\alpha$ is the intercept, and $\epsilon _t$ is the residual (also referred to as random shock or innovation) at time $t$, with $\epsilon _{t} \sim \mathcal {N}(0,4)$. Now, the equilibrium in the AR model is also equal to the mean of the time series, similar to how the resting position of a pendulum is both the equilibrium and mean of the pendulum’s positions over time. Therefore, we can center the data by subtracting the equilibrium $\mu$ (which is 15 for both persons), and this makes the intercept zero while the $\phi$ parameter is unchanged. Moving $\mu$ to the right side, we obtain

1 $$\begin{aligned} y_{t} =\mu + \phi (y_{t-1} -\mu ) + \epsilon _{t}. \end{aligned}$$

It follows that the intercept term $\alpha$ in the uncentered model is not equal to the equilibrium $\mu$ of the person, but rather $\alpha = (1-\phi ) \mu$. Since the intercept term has no intuitive interpretation, we prefer the centered model notation of Eq. 1, which includes $\mu$, rather than $\alpha$.

The autoregressive coefficient $\phi$ should lie between $-$1 and 1 to obtain a stationary time series with constant variance (e.g., Hamilton, 1994). From the equations above, it can be seen that a $\phi$ value close to 0 implies that there is little to no carry-over from one moment to the next; therefore, even after an extreme score, the person will quickly recover to their equilibrium. In contrast, a positive $\phi$ value closer to 1 implies more carry-over from one moment to the next, reflecting regulatory weakness. Negative values of $\phi$ have a different interpretation, because they imply reflexive back-and-forth shifting between scores above and below the equilibrium. Since this does not seem plausible in the context of affect regulation, we focus on positive $\phi$ values here [but see Rovine and Walls (2006) for an example of a process with a negative $\phi$ value].

### The Multilevel AR Model

When time series for multiple persons are available, we can create a multilevel AR model by specifying that the inertia parameters and equilibria of the persons can take different values, but that they come from a common distribution. Extending Eq. 1 with subject-indices and a normal density for the inertias and equilibria, the following multilevel model is obtained:

2 $$\begin{aligned} \begin{array}{rcl} Y_{t,i} &{} = &{} \mu _i + \phi _i (Y_{t-1,i} -\mu _i ) + \epsilon _{t,i}, \\ \epsilon _{t,i} &{} \sim &{} \mathcal {N} (0,\sigma ^2_{\epsilon }), \\ \phi _i &{} = &{} \gamma _{\phi } + u_{\phi ,i}, \\ \mu _i &{} = &{} \gamma _\mu + u_{\mu ,i} , \\ \begin{bmatrix} u_{\phi ,i} \\ u_{\mu ,i} \\ \end{bmatrix} &{} \sim &{} \mathcal {N} \left( \begin{bmatrix} 0 \\ 0 \end{bmatrix} , \begin{bmatrix} \sigma ^2_{\phi } &{} \\ \sigma _{\phi \mu } &{} \sigma ^2_{\mu } \\ \end{bmatrix} \right) , \end{array} \end{aligned}$$

where $\gamma _{\phi }$ represents the average inertia, and $\gamma _\mu$ represents the average equilibrium.

This multilevel AR model enables researchers to estimate the average inertia in the population and to use observed person-level variables as predictors for the inertias, to see which person characteristics are related to regulatory weakness. This is the approach that was taken by Suls et al. (1998) and Kuppens et al. (2010), who showed that regulatory weakness is indeed related to person characteristics such as depression and neuroticism.

### The TAR Model for a Single Person

While the inertia parameter $\phi$ in the AR(1) model has been interpreted as a measure of a person’s regulatory weakness, the experimental study by Koval and Kuppens (2011) shows that it may not be appropriate to treat emotional inertia as a fixed trait of an individual. We expect that in reality, inertia varies with the intensity of affect experienced at a given time. Furthermore, it makes sense to distinguish between inertia for increased and decreased affect, for reasons of interpretation: While high inertia during intense negative affect can be considered maladaptive, high inertia during an episode of (relative) absence of negative affect may actually be considered adaptive, since it characterizes a person who experiences more prolonged periods of little negative affect. For this reason, we argue that it is important to consider the inertias for decreased and increased affect separately, especially when studying relationships with psychological disorders or personality traits.

Our hypothesis of state-dependent affect regulation can be represented nicely by a TAR(2,1,1) model (Tong & Lim, 1980), which is based on two AR(1) processes; at each occasion, one of these processes generates the data. The notation TAR(2,1,1) indicates that there are two alternating AR processes, and that both of them have auto-regression only at lag 1; hereafter, we will simply refer to this specific model as the TAR model. Technically, the model that we propose distinguishes between episodes of increased and decreased affect by using a different $\phi$ coefficient depending on the value of the previous observation: If this value is smaller than the *threshold* value $\tau$, then the model uses the first $\phi$ coefficient, but if the previous observation is larger than $\tau$, the other $\phi$ coefficient applies. Throughout this article we will refer to these two states of the model as the “lower state” and “upper state,” respectively. In this model, the threshold $\tau$ can be thought of as the equilibrium, the value of negative affect that separates the states of *decreased* and *increased* negative affect. Just like the AR model, the TAR model predicts that when a person’s negative affect is changed by some external event, it will, eventually, return to the equilibrium. However, in the TAR model, the recovery from increased and decreased negative affect will not necessarily happen equally quickly. Going back to the pendulum analogy, the TAR process could be compared to a pendulum that swings further or more often to one side than to the other, despite having the same resting position. As a result, the equilibrium in the TAR model does *not* equal the mean of the time series, as it did in the AR model.

To see what kind of pattern the TAR model may generate, consider the negative affect scores of the hypothetical person C, which are depicted in the bottom panel of Figure 1. These scores were simulated under a TAR model with $\phi =0.1$ in the lower state and $\phi =0.7$ in the upper state. The equilibrium of C is $\tau =15$ and is depicted by the horizontal line. Although it may not be immediately obvious, closer inspection of the time series reveals the asymmetry around the equilibrium: When person C’s negative affect is less intense ($<$15), recovery to the equilibrium occurs quickly due to the lower inertia in this state, but when his/her negative affect is more intense ($>$15), it takes longer to recover as a result of the higher inertia. Because of this asymmetry, the mean observed score of person C is 16, which is somewhat higher than the equilibrium of 15. The state-space plot for person C (the lower right panel of Figure 1) shows that the autoregressive relationship is stronger for affect scores above the equilibrium, illustrating that person C is characterized by weaker affect regulation for increased negative affect. This kind of affect regulation could be considered rather maladaptive.

The TAR model equation underlying person C’s affect scores is given by

3 $$\begin{aligned} y_{t} = \left\{ \begin{array}{rl} \tau + \phi _1 (y_{t-1} - \tau ) + \epsilon _{t} &{}\quad \text {if } \qquad y_{t-1} < \tau \\ \tau + \phi _2 (y_{t-1} - \tau ) + \epsilon _{t} &{} \quad \text {if }\qquad y_{t-1} \ge \tau \end{array}\right\} , \end{aligned}$$

with parameter values $\tau =15$, $\phi _1=0.1$, $\phi _2=0.7$, and $\epsilon _{t} \sim \mathcal {N} (0,4)$. As in the AR model, the $\phi$ coefficients should have values smaller than 1 to obtain a stationary process. Note that we have immediately written the model for equilibrium-centered data, similar to Eq. 1 for the AR model. In that model, we centered the scores around the equilibrium, which equaled the mean, so that the intercept ($\alpha$) became zero. However, in the current TAR model, the equilibrium $\tau$ does not equal the mean, so that centering around the equilibrium does not automatically make the intercepts zero. There is another reason here why we center the data around the equilibrium and remove the intercepts: We want the two regression lines in the TAR model to connect at the threshold, as they do in the state-space plot for person C in Figure 1. Centering the scores around the threshold and forcing the intercepts to be zero ensures that this is the case. This model restriction serves to make the model substantively appropriate, because it ensures that a person’s affect is always *predicted* to return to their equilibrium level of affect. In contrast, if the regression lines did not connect, this discontinuity in the model would have a highly unrealistic implication, namely a sudden shift in the predicted affect *away* from the equilibrium whenever the previous observation happened to be close to it (Madhyastha, Hamaker, & Gottman, 2011). Note that the residual innovation $\epsilon$ always allows random shifts in the actual observed scores.

### The Multilevel TAR Model

In this study, we propose to extend the TAR model of Eq. 3 so that data from multiple individuals can be analyzed at once, treating $\tau$, $\phi _1$ and $\phi _2$ as random effects. Thus, each person can have a different threshold value $\tau$, representing their equilibrium that separates the two states. In addition, the inertias during episodes of decreased and increased affect can vary between persons. The thresholds and the inertias can be modeled by a multivariate normal distribution at the between-persons level, giving us this model:

4 $$\begin{aligned} \begin{array}{l} Y_{t,i} = \left\{ \begin{array}{rl} \tau _i + \phi _{1,i} (Y_{t-1,i} - \tau _i) + \epsilon _{t,i} &{}\qquad \text {if } \qquad Y_{t-1,i} < \tau _i\\ \tau _i + \phi _{2,i} (Y_{t-1,i} - \tau _i) + \epsilon _{t,i} &{} \qquad \text {if }\qquad Y_{t-1,i} \ge \tau _i \\ \end{array} \right\} , \\ \begin{array}{ccl} \epsilon _{t,i} &{} \sim &{} \mathcal {N} (0,\sigma ^2_{\epsilon }), \\ \phi _{1,i} &{} = &{} \gamma _{\phi _1} + u_{\phi _1,i} , \\ \phi _{2,i} &{} = &{} \gamma _{\phi _2} + u_{\phi _2,i} , \\ \tau _{i} &{} = &{} \gamma _{\tau } + u_{\tau ,i}, \\ \begin{bmatrix} u_{\phi _1,i} \\ u_{\phi _2,i} \\ u_{\tau ,i} \\ \end{bmatrix} &{} \sim &{} \mathcal {N} \left( \begin{bmatrix} 0 \\ 0 \\ 0 \end{bmatrix} , \begin{bmatrix} \sigma ^2_{\phi _1} &{} &{} \\ \sigma _{\phi _2 \phi _1} &{} \sigma ^2_{\phi _2} &{} \\ \sigma _{\tau \phi _1 } &{} \sigma _{\tau \phi _2} &{} \sigma ^2_{\tau } \end{bmatrix} \right) , \end{array} \end{array} \end{aligned}$$

where the fixed effects $\gamma$ represent the average inertias and threshold over persons, and the random effects can be correlated.

Like the multilevel AR model, this multilevel TAR model takes into account interpersonal differences in regulatory weakness. However, unlike the AR model, this model also takes into account that regulatory weakness varies with the intensity of affect. Since the model lets each person have their own threshold parameter, the lower and upper state are always relative to the person’s own equilibrium. Importantly, using the multilevel TAR model, researchers can use person-level variables as predictors both for the inertias, representing the state-dependent regulatory weakness, and for the threshold representing a person’s equilibrium. This particular advantage of the model will be illustrated later on, in the empirical applications.

## Simulations

We performed simulations to investigate how well we can distinguish between TAR and AR processes in terms of power and Type I error. We generated data under both models using R (R Development Core Team, 2012) and estimated the TAR model of Eq. 4 in OpenBUGS, an open-source program for Bayesian model estimation (Lunn, Spiegelhalter, Thomas & Best, 2009). In this framework, all the parameters of the multilevel TAR model can be estimated simultaneously, and the model specifications are straightforward.

Bayesian estimation is used here because classical approaches are problematic for the multilevel TAR model. In standard multilevel software, it is not even possible to specify the multilevel TAR model unless plugin values are used for the unknown thresholds. We did some preliminary analyses substituting the individual means for the thresholds, and estimating the models using the *lme* package in R (Bates, Maechler, & Bolker, 2011; R Development Core Team, 2012), but the method performed so poorly^1^ that we did not consider it a viable approach. Other possibilities within the frequentist framework would be to use estimation procedures based on the first-order Taylor expansion method or adaptive Guassian quadrature: Wang and McArdle (2008) used these for estimating the closely related (Chen, 1998; Hamaker, 2009) multilevel changepoint model. However, their results indicated that the estimates were highly dependent on the specified starting values, which makes these estimation methods unattractive. In contrast, Bayesian estimation was shown to lead to good results (Wang & McArdle, 2008), and we decided to take this approach, which has the additional advantage that the thresholds can be modeled and predicted from other variables.

### Data Generation

We simulated negative affect data from three multilevel TAR models and one multilevel AR model. From each of these four models we drew random samples of varying sizes. The number of persons (*N*) ranged from 50 to 150 (in steps of 25), and the number of observations (or time points, *T*) per person was either 50, 100, 150 or 200. Combining these two factors resulted in twenty conditions. For each condition, one hundred samples were simulated.^2^

In the AR model, the inertia $\phi _i$ was normally distributed over persons with $\gamma _{\phi } = 0.4$ and $\sigma _{\phi } = 0.1$. In the TAR models, the fixed effects $[\gamma _{\phi _1}, \gamma _{\phi _2}]$ were $[0.2, 0.3]$, $[0.2, 0.4]$, or $[0.2, 0.5]$. The standard deviations $\sigma _{\phi _1}$ and $\sigma _{\phi _2}$ were always 0.1. The threshold $\tau$ in all three TAR models was distributed normally with $\gamma _{\tau } = 15$ and $\sigma _{\tau } = 2$. In the TAR models, the correlation between the inertias, $\rho _{\phi _1 \phi _2}$, was set to 0.5, because we expect that people who have weaker affect regulation during episodes of increased negative affect will also tend to have somewhat weaker regulation during episodes of decreased negative affect, and vice versa. The level-1 innovation variance $\sigma ^2_{\epsilon }$ was set to 4 in both the TAR and AR models.

If we consider the effect sizes of the mean inertia differences implied by the three TAR settings, they correspond to Cohen’s $d$ values of 1, 2, and 3, respectively. These are very large effect sizes, but the choices for the means and standard deviations of the inertia parameters were based on substantive considerations, i.e., on which parameter values seem realistic for affect regulation. Smaller effect sizes could be obtained by setting a higher value for $\sigma _{\phi }$ or specifying a smaller difference between $\gamma _{\phi _1}$ and $\gamma _{\phi _2}$. However, setting a larger standard deviation would imply that many persons have negative $\phi _1$ values, which we consider unrealistic for reasons described above, while smaller differences between the average inertias than 0.1 (the smallest difference we chose) do not seem clinically relevant to us. Therefore, we decided on these parameter values by considering the unique interpretation and scale of the inertia parameters in the TAR model, rather than focusing on measures of effect size.

### Estimation

We estimated the model from Eq. 4, noting that this particular model formulation (i.e., using zero intercepts and centering the data around the estimated threshold) not only ensures that the model has connecting regression lines, but in addition, it helps to identify the threshold parameter for each person in the sample, even if their estimated inertias happen to be equal for increased and decreased affect (normally a problematic situation, confer Hansen, 1996; Andrews & Cheng, 2012). The BUGS input files for the multilevel AR and TAR models are given in Appendix 1.

We wanted vague prior parameter densities because we aimed for data-driven model estimates. The multivariate normal prior density of the level-2 parameters had a mean vector of zeros and a precision matrix (the inverse of the covariance matrix) with diagonal elements equal to 1.0E$-$6 and off-diagonal elements equal to 0. For the inverse of the covariance matrix for the inertias and thresholds, the prior density was a Wishart distribution using an identity matrix. For the inverse of the level-1 residual variance, we specified a gamma density with the shape and scale parameters 0.001 and 0.001. It must be noted that the inverse gamma and Wishart priors for variance parameters have been criticized because they may actually bias (co)variance estimates (Gelman, 2006), but they are oft-used and recommended proper priors in OpenBUGS for which a better alternative is not yet available to our knowledge. If the results of our simulations are satisfactory using these suboptimal (co)variance priors, the analytical results in future applications with better priors should only be even more reliable.

To speed up the convergence of the MCMC sampling algorithm, we specified starting values of a realistic magnitude (see Appendix 1). A burn-in period of 1,000 iterations was used, and 2,000 iterations were used for inference. Convergence can be assessed in OpenBUGS by running two chains, and inspecting the trace and history plots, autocorrelations, and Brooks–Gelman–Rubin diagnostics for all parameters (at level 1 and 2). Using this approach to inspect the convergence for a random subset of the fitted models, we concluded that this number of burn-in iterations was enough for the models to reach convergence.

### Results

In keeping with our purpose of determining how well we can distinguish between TAR and AR processes, we formulated a decision criterion for choosing between the AR and TAR models for each sample. This was followed by calculation of the *power* to detect the TAR process, and of the Type I error rates (i.e., the proportion of AR samples where we erroneously selected the TAR model). In addition, we inspected the width and actual coverage rate of the 95 % credible intervals of the level-2 parameters $\gamma _{\phi _1}, \gamma _{\phi _2},$ and $\gamma _{\tau }$. Bayesian credible intervals have a different interpretation than classical confidence intervals because they are not defined in terms of frequentist coverage, but in terms of the researcher’s (possibly subjective) posterior certainty about the parameter. For this reason, Bayesian credible intervals can be constructed for functions or combinations of parameters that are difficult to handle in the frequentist setting, but it also means that the frequentist coverage of a 95 % Bayesian credible interval does not always have to be equal to .95 (this depends on the specified priors, among other things). When the coverage approximates this value, however, we can interpret the credible interval and use it as a decision criterion without necessarily adopting a fully Bayesian perspective.

As a criterion for model selection, we first considered the Deviance Information Criterion (DIC; Spiegelhalter, Best, Carlin, & Van Der Linde, 2002), which is easily obtained in OpenBUGS. However, the DIC is known to be problematic for mixture-type models (cf. Celeux, Forbes, Robert, & Titterington, 2006; Spiegelhalter et al., 2002) and our analyses showed that the power to detect the TAR process with the largest effect size (i.e., when $[\gamma _{\phi _1}, \gamma _{\phi _2}] = [0.2, 0.5]$), was almost zero when using the DIC. Model comparison using the Bayes Factor would be more involved, and is sensitive to the choice of prior distributions (Song & Lee, 2012). Therefore, we decided upon a different, intuitive model selection criterion directly involving the average difference in inertia between the two states. We included the quantity $( \gamma _{\phi _2} - \gamma _{\phi _1})$ in our TAR model syntax, so that, in each iteration of the MCMC sampler, OpenBUGS calculated this quantity based on the current draws of $\gamma _{\phi _1}$ and $\gamma _{\phi _2}$. Thus, a posterior distribution was obtained for this difference, and the 95 % credible interval of this difference was then used as a decision criterion: When 0 was included in the credible interval, there was no evidence that there are *two different* mean inertias, so we selected the multilevel AR model; when 0 was *not* included in the credible interval of the mean difference, this was taken as evidence that there are two distinct states with different mean inertias, so we selected the multilevel TAR model.

#### Type I Error and Power

Using the decision criterion described above, the Type I error rate for each sample size was well below the conventional level of .05 for classical hypothesis tests, as can be seen in Table  1. When the TAR model had actually generated the data, our interest was in the detection rates (i.e., power) and these are given in Table 2. The multilevel TAR model with the smallest effect size (i.e., when $[\gamma _{\phi _1}, \gamma _{\phi _2}] = [0.2, 0.3]$) could be detected with adequate power ($>$.80) in some of the larger sample sizes under consideration. The multilevel TAR models with larger effect sizes could be detected with high power ($\ge$ .90) even in the smallest sample sizes that were used (i.e., $N=50$ and $T=50$).

**Table 1:** Type I error rates for each set of 100 AR samples, using the 95 % credible interval decision criterion.

**N**	*T:*	*50*	*100*	*150*	*200*
**50**		**.02**	**.01**	**.00**	**.01**
**75**		**.01**	**.00**	**.01**	**.00**
**100**		**.02**	**.00**	**.00**	**.00**
**125**		**.01**	**.01**	**.00**	**.00**
**150**		**.01**	**.02**	**.01**	**.00**

A Type I error was made when the 95 % credible interval of ($\gamma _{\phi _2} - \gamma _{\phi _1}$) did not include zero, so that we selected the TAR model, incorrectly. Acceptable Type I error rates ($<$.05) are bold-faced.

**Table 2:** Power rates for each set of 100 samples using the 95 % credible interval decision criterion.

		$\gamma \phi = [0.2, 0.3]$				$\gamma \phi = [0.2, 0.4]$				$\gamma \phi = [0.2, 0.5]$			
**N**	*T:*	*50*	*100*	*150*	*200*	*50*	*100*	*150*	*200*	*50*	*100*	*150*	*200*
**50**		.08	.17	.27	.36	.58	**.94**	**.95**	**1**	**.90**	**1**	**1**	**1**
**75**		.19	.37	.58	.67	**.84**	**1**	**.99**	**1**	**1**	**1**	**1**	**1**
**100**		.30	.55	**.81**	**.90**	**.94**	**.99**	**1**	**1**	**1**	**1**	**1**	**1**
**125**		.41	.79	**.90**	**.99**	**.98**	**1**	**1**	**1**	**1**	**1**	**1**	**1**
**150**		.49	**.82**	**.97**	**.98**	**.99**	**1**	**1**	**1**	**1**	**1**	**1**	**1**

The multilevel TAR model was selected, correctly, when the 95 % credible interval of ($\gamma _{\phi _2} - \gamma _{\phi _1}$) did not include zero. Adequate power values ($>$.80) are bold-faced.

#### Accuracy of the Level-2 Estimates

As point estimates of the level-2 parameters we used the means of their posterior distributions. Table 3 gives the absolute bias of these estimates (i.e., estimate – true value), as well as the width and actual coverage of their 95 % credible intervals. It can be seen that the mean threshold and the mean inertias are estimated accurately, with (approximately) adequate 95 % credible interval coverage for all sample sizes. The only exception is the 95 % credible interval of $\gamma _{\phi _2}$, which tends to have insufficient coverage when the sample includes many persons (decreasing the width of the interval) and few measurements per person.

**Table 3:** Bias of the point estimates (i.e., the posterior means) for the average inertias and threshold, and coverage and width of their 95 % credible intervals, based on 100 fitted models per sample size.

			Bias				Coverage				Width			
$\gamma _{\phi } = [0.2, 0.3]$	**N**	*T:*	*50*	*100*	*150*	*200*	*50*	*100*	*150*	*200*	*50*	*100*	*150*	*200*
$\gamma _{\phi _1}$	**50**		$-$.03	$-$.02	$-$.01	$-$.01	98	100	98	99	.23	.18	.16	.15
	**75**		$-$.02	$-$.02	$-$.01	$-$.01	99	98	97	98	.18	.14	.12	.11
	**100**		$-$.01	$-$.01	$-$.01	$-$.01	97	98	96	97	.15	.12	.10	.09
	**125**		$-$.02	$-$.02	$-$.01	$-$.01	93	96	96	98	.14	.10	.09	.08
	**150**		$-$.01	$-$.01	$-$.01	$-$.01	99	93	97	95	.12	.09	.08	.07
$\gamma _{\phi _2}$	**50**		$-$.04	$-$.02	$-$.02	$-$.02	96	98	99	98	.22	.17	.15	.14
	**75**		$-$.03	$-$.02	$-$.01	$-$.01	98	97	97	97	.17	.13	.11	.10
	**100**		$-$.03	$-$.02	$-$.01	$-$.01	91	96	96	97	.14	.11	.09	.09
	**125**		$-$.02	$-$.01	$-$.01	$-$.01	96	98	97	98	.12	.09	.08	.07
	**150**		$-$.02	$-$.02	$-$.01	$-$.01	91	93	98	96	.11	.08	.07	.07
$\gamma _{\tau }$	**50**		.00	.01	.01	.00	95	94	91	93	1.19	1.17	1.15	1.14
	**75**		.03	.03	.00	.03	96	94	97	92	.97	.94	.93	.92
	**100**		.03	$-$.01	.01	.03	95	95	95	94	.83	.81	.80	.80
	**125**		.01	.02	.01	.04	97	97	94	95	.74	.73	.72	.72
	**150**		.02	.02	.02	.01	95	93	95	97	.68	.66	.65	.65
$$\gamma _{\phi } = [0.2, 0.4]$$														
$\gamma _{\phi _1}$	**50**		$-$.03	$-$.02	$-$.01	$-$.01	99	98	100	99	.24	.19	.16	.15
	**75**		$-$.02	$-$.01	$-$.01	$-$.01	96	96	99	96	.18	.14	.12	.12
	**100**		$-$.01	$-$.01	$-$.01	$-$.01	99	99	97	98	.15	.12	.10	.10
	**125**		$-$.02	$-$.02	$-$.01	$-$.01	90	97	98	95	.14	.11	.09	.08
	**150**		$-$.02	$-$.01	$-$.01	$-$.01	94	93	97	96	.12	.09	.08	.07
$\gamma _{\phi _2}$	**50**		$-$.03	$-$.01	$-$.01	$-$.01	95	99	100	100	.20	.16	.14	.13
	**75**		$-$.03	$-$.02	$-$.01	$-$.01	94	97	99	99	.15	.12	.11	.10
	**100**		$-$.03	$-$.02	$-$.01	$-$.01	90	96	95	97	.13	.10	.09	.08
	**125**		$-$.02	$-$.02	$-$.01	$-$.01	92	95	94	97	.11	.09	.08	.07
	**150**		$-$.03	$-$.02	$-$.01	$-$.01	86	94	96	99	.10	.08	.07	.06
$\gamma _{\tau }$	**50**		.02	.01	.07	.04	93	96	91	92	1.20	1.16	1.15	1.14
	**75**		.05	.00	.03	$-$.02	94	98	95	95	.96	.95	.93	.93
	**100**		.05	.00	.02	.03	94	96	95	99	.82	.82	.81	.79
	**125**		$-$.01	$-$.02	.03	$-$.01	95	96	96	97	.75	.73	.72	.72
	**150**		.02	.03	.00	.01	94	88	95	93	.69	.66	.66	.65
$$\gamma _{\phi } = [0.2, 0.5]$$														
$\gamma _{\phi _1}$	**50**		$-$.04	$-$.02	$-$.01	$-$.01	97	96	100	99	.26	.20	.17	.16
	**75**		$-$.02	$-$.01	$-$.01	$-$.01	97	99	99	100	.20	.15	.13	.12
	**100**		$-$.01	$-$.01	$-$.00	$-$.01	100	97	99	99	.17	.12	.11	.10
	**125**		$-$.01	$-$.01	$-$.01	$-$.01	98	99	95	96	.15	.11	.09	.09
	**150**		$-$.02	$-$.01	$-$.01	$-$.01	98	96	94	98	.13	.10	.08	.08
$\gamma _{\phi _2}$	**50**		$-$.03	$-$.03	$-$.02	$-$.01	96	98	100	100	.19	.15	.14	.13
	**75**		$-$.04	$-$.02	$-$.02	$-$.02	93	98	97	98	.15	.11	.10	.09
	**100**		$-$.03	$-$.02	$-$.01	$-$.01	88	97	99	98	.12	.09	.08	.08
	**125**		$-$.03	$-$.02	$-$.01	$-$.01	80	93	96	98	.11	.08	.07	.07
	**150**		$-$.03	$-$.01	$-$.01	$-$.01	83	92	92	96	.09	.07	.06	.06
$\gamma _{\tau }$	**50**		.07	.03	.00	.00	95	95	96	98	1.21	1.16	1.14	1.15
	**75**		.03	$-$.01	.01	.00	95	98	92	90	.99	.95	.93	.92
	**100**		.05	.04	.02	.03	93	93	97	97	.85	.83	.80	.81
	**125**		.04	.03	.01	.00	95	97	97	95	.76	.73	.73	.72
	**150**		.01	.02	.01	.00	96	94	98	94	.69	.67	.66	.65

Negative values indicate underestimation. Absolute values below .005 are rounded to .00.

With regard to the width of the 95 % credible intervals, we note that the intervals shrink considerably when the number of persons is increased, but they are less affected when the number of time points is increased. We can understand these differential influences if we consider that these 95 % credible interval are for the level-2 parameters, so they represent the uncertainty about the *average* inertia or threshold in the population. When more persons are included in the study, we logically gain more certainty about this average parameter so that the width of the interval decreases. In contrast, while increasing the number of time points does help to get more accurate estimates and smaller 95% credible intervals for the level-1 parameters (i.e., for the individual inertias and thresholds), it does not add that much information with regard to the certainty about the population mean. These considerations can be used to weigh the costs and benefits of increasing the sample size at level 1 or at level 2, depending on the researcher’s primary goals: If the interest is mostly in the average parameters, increasing the number of persons is more useful, but if the focus is more on the individual estimates it becomes more important to use a large number of measurements.

### Conclusion

Based on the results of our simulations, we can conclude that Bayesian estimation of the multilevel TAR model is feasible for the sample sizes under consideration, and yields accurate estimates of the average inertias and threshold. When we want to distinguish between an AR process and a TAR process, a decision criterion based on the 95 % credible interval of the difference in mean inertias $(\mu _{\phi _2} - \mu _{\phi _1})$ gives us adequate power and minimal Type I error. Model selection using the DIC proved to be much less powerful and is not recommended for this purpose.^3^

## Empirical Applications

### Application 1: Moment-to-Moment Affective Behavior of Spouses

**Fig. 2:**
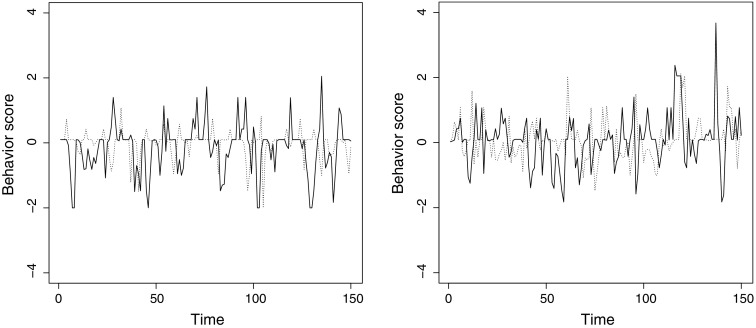
Plotted scores for the affective behavior of two example couples during the conflict discussion task. The *solid line* connects the wife’s scores, the *dotted line* the husband’s scores.

After concluding that Bayesian estimation of the multilevel TAR model works well, we applied this modeling approach to an empirical data set concerning the affective behavior of 129 newlywed couples during a 15-min conflict discussion (Gottman, Swanson, & Murray, 1999; see also Gottman, Murray, & Swanson, 2005). Each spouse’s affective behavior (including nonverbal behavior) was coded per second by two independent observers using the specific affect coding system (SPAFF; Gottman & Krokoff, 1989). The scores were then averaged over 6-s intervals and across both coders, resulting in $T = 150$ observations for each spouse. In this data set, negative scores and scores between 0 and 0.1 indicated negative behavior (e.g., anger or contempt), and positive scores greater than 0.1 indicated friendly or constructive behavior (e.g., humor or validation). The score 0.1 corresponded to neutral behavior, or to positive and negative behavior canceling out within the 6-s interval. In Figure 2, the data of some of the couples are portrayed in a time series plot. Since we detected no gross violations of stationarity (i.e., time trend) we used the raw data of the persons without applying differencing or detrending techniques (e.g., Hamilton, 1994).

Madhyastha et al. (2011) analyzed these data using single-person TAR and AR models for each spouse, rather than a multilevel model. In their approach, the focus was on the way spouses influenced each other (and whether or not this influence was state-dependent), while inertia was always modeled as a fixed trait of the person. Here, we use the multilevel TAR model to investigate whether inertia was actually state-dependent, while also taking into account interpersonal differences in the regulation of affective behavior.

#### Hypotheses

When fitting a multilevel autoregressive model to these behavioral scores, the inertia parameter of a spouse reflects how they regulated their affective behavior during the conversation. The multilevel TAR model allows the inertia parameter to be state-dependent, such that the strength of regulation could differ between (more) positive and (more) negative behavior. As in our simulations, the 95 % credible interval of the level-2 inertia difference was used to decide whether the regulation of affective behavior was, on average, state-dependent.

To take into account the dyadic structure in this data set, we could have specified two related TAR models, one for the husbands and one for the wives. However, since the spouses were observed while interacting, it makes more substantive sense to extend our multilevel TAR model to a bivariate version, wherein the husband and wife are seen as a bivariate system with mutual influence and correlated inertias and thresholds. This model can be thought of as a multilevel threshold-vector autoregressive (VAR) model, because it extends the multilevel VAR model by letting the autoregressive coefficients depend on the threshold variables. In this model, each dyad had four inertia parameters, namely two for each spouse, and a different threshold parameter for each spouse, as well as two influence parameters representing the lagged effect of the husband’s behavior in the previous interval on the wife’s behavior in the current interval, and vice-versa.

We also included the correlation at the dyad level between husbands’ and wives’ inertias and thresholds in the model, to take into account possible similarity between spouses in their regulation of affective behavior. Furthermore, the model allowed us to address an additional research question, namely, whether there is a gender difference in the regulation of affective behavior. This question could be investigated by subtracting the mean inertias and threshold of the men from those of the women and obtaining 95 % credible intervals for each of the differences. If zero is not included in such a difference interval, it can be taken as evidence of a gender difference with regard to that parameter.

Writing $H_{t,i}$ for the score of the husband of couple $i$ at time point $t$, the model for the husband is given by:

$$\begin{aligned} \begin{array}{l} H_{t,i}\!=\! \left\{ \!\begin{array}{rl} \tau _{H,i} + \phi _{1,H,i} (H_{t-1,i} - \tau _{H,i}) + \beta _{H,i} (W_{t-1,i} - \tau _{W,i}) + \epsilon _{H,t,i} &{}\qquad \text {if } \qquad H_{t-1,i} < \tau _{H,i}\\ \tau _{H,i} + \phi _{2,H,i} (H_{t-1,i} - \tau _{H,i}) + \beta _{H,i} (W_{t-1,i} - \tau _{W,i}) + \epsilon _{H,t,i} &{} \qquad \text {if }\qquad H_{t-1,i} \ge \tau _{H,i} \\ \end{array}\! \right\} \!, \\ \begin{array}{ccl} \epsilon _{H,t,i} &{} \sim &{} \mathcal {N} (0,\sigma ^2_{\epsilon _H}), \\ \phi _{1,H,,i} &{} = &{} \gamma _{\phi _{1,H}} + u_{\phi _{1,H},i} , \\ \phi _{2,H,i} &{} = &{} \gamma _{\phi _{2,H}} + u_{\phi _{2,H},i} ,\\ \tau _{H,i} &{} = &{} \gamma _{\tau _{H}} + u_{\tau _H,i}, \\ \beta _{H,i} &{} = &{} \gamma _{\beta _{H}} + u_{\beta _{H,i}} ,\end{array} \end{array} \end{aligned}$$

and similarly for the wife. The eight random effects (four inertias, two thresholds, two influence parameters $\beta$) are multivariate normally distributed with mean vector and covariance matrix to be estimated. The BUGS syntax for estimating this model is given in Appendix 2.

#### Results

We ran the model with two parallel MCMC chains using different starting values. We used 20,000 burnin iterations, after which the convergence was adequate and 50,000 samples (per chain) were used for inference. With this number of iterations, the analysis took 3 h and 6 min on a system with 16 GB RAM, running on a single core with 3.40 GHz processing speed.

**Fig. 3:**
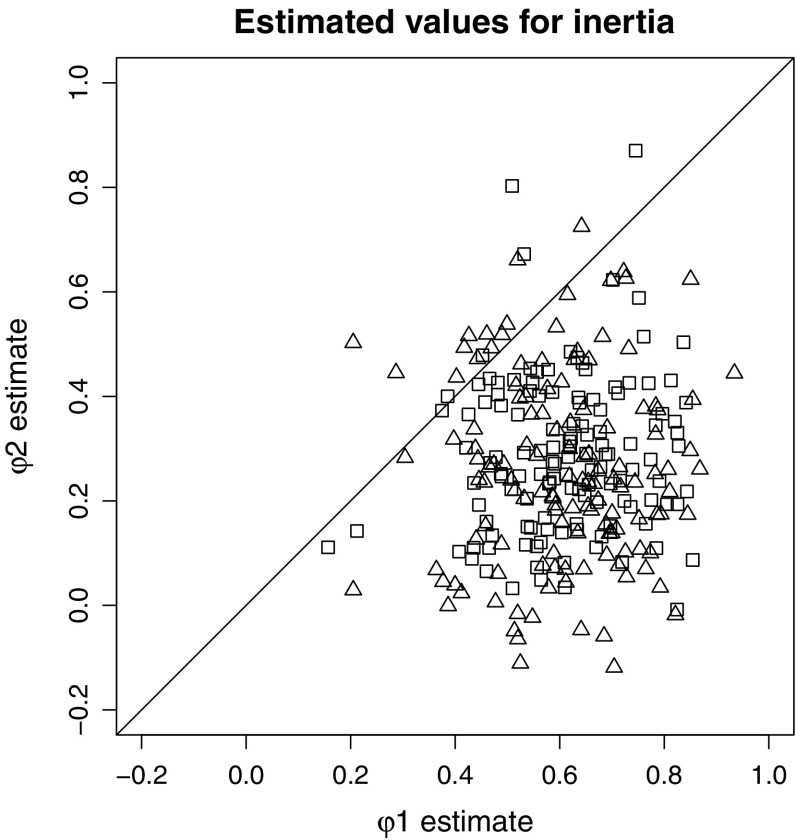
Scatterplot of the estimated level-1 inertias in the more negative state ($\phi _1$) and the more positive state ($\phi _2$). Each *square* represents a husband and each *triangle* represents a wife. Since most of them fall below the *diagonal* (equality) *line*, we can conclude that the majority of the spouses was characterized by weaker regulation during episodes of more negative affective behavior.

Table 4 summarizes the results of the model parameters of interest. The credible interval of ($$\gamma _{\phi _2} - \gamma _{\phi _1}$$) in the TAR model was $$[ -0.39, -0.25]$$ for the husbands and $$[ -0.42, -0.27]$$ for the wives. Since neither of these intervals included zero, we concluded that, on average, the inertia of both the husbands and the wives was state-dependent. The point estimate (posterior mean) of the mean inertia in the lower state (i.e., for more negative affective behavior) was $$0.61$$ for husbands and $$0.60$$ for wives, while the mean inertia in the upper state (i.e., for more positive affective behavior) was $$0.29$$ for husbands and $$0.26$$ for wives. The mean thresholds were $$\gamma _{\tau _{H}} = 0.07$$ and $$\gamma _{\tau _{W}} = 0.08$$, so that for both husbands and wives the two states (almost) coincided with the negative and positive ranges of the SPAFF behavior coding system (since 0.1 represents the neutral score). These results indicated that, on average, the regulation of negative affective behavior was weaker than the regulation of positive affective behavior. Figure 3 depicts the level-1 estimates for all persons in the sample, plotting the inertia in the more positive state ($$\phi _2$$) against the inertia in the more negative state ($$\phi _1$$). From this plot we can see that our conclusion, based on the level-2 estimates, holds for most of the individuals: They had higher inertia during more negative affective behavior (i.e., $$\phi _1 > \phi _2$$). Using the estimates of the (co-)variances of the inertia coefficients over persons we can also calculate the effect size (just as we did for our simulated data) and we obtain Cohen’s $$d = 3.9 $$ for the men and $$d = 20 $$ for the women.

**Table 4:** Point estimates (posterior means) and 95 % credible intervals for selected parameters of the bivariate TAR model in application 1.

	Mean	95 % CCI
Husbands		
$\gamma _{\phi _{1,H}}$	0.61	[0.57,0.65]
$\gamma _{\phi _{2,H}}$	0.29	[0.24, 0.34]
$(\gamma _{\phi _{2,H}} - \gamma _{\phi _{1,H}})$	$-$0.32	[$-$0.39, $-$0.25]
$\gamma _{\tau _{H}}$	0.07	[0.006, 0.139]
$\gamma _{\beta _{H}}$	0.007	[$-$0.020, 0.035]
Wives		
$\gamma _{\phi _{1,W}}$	0.60	[0.56, 0.64]
$\gamma _{\phi _{2,W}}$	0.26	[0.20, 0.31]
$(\gamma _{\phi _{2,W}} - \gamma _{\phi _{1,W}})$	$-$0.35	[$-$0.42, $-$0.27]
$\gamma _{\tau _{W}}$	0.08	[0.01, 0.15]
$\gamma _{\beta _{W}}$	0.018	[$-$0.007, 0.044]
Gender comparisons		
$\gamma _{\phi _{1,W}} - \gamma _{\phi _{1,H}}$	$-$0.007	[$-$0.056, 0.043]
$\gamma _{\phi _{2,W}} - \gamma _{\phi _{2,H}}$	$-$0.03	[$-$0.11, 0.04]
$\gamma _{\tau _{W}} - \gamma _{\tau _{H}}$	0.007	[$-$0.059,0.071]
Spouse correlations		
$r_{\phi _{1}}$	0.25	[0.05, 0.45]
$r_{\phi _{2}}$	0.09	[$-$0.15,0.33]
$r_{\tau }$	0.60	[0.46, 0.73]

Based on the credible intervals we conclude that inertia was, on average, state-dependent for both the men and women. There were no gender differences in affect regulation, but some correlation between the model parameters for spouses.

To investigate gender differences in the regulation of affective behavior, we inspected the 95 % credible intervals of the gender differences in the two mean inertias and in the mean threshold. Since all three intervals included zero, there was no indication of any gender difference. We also found no evidence of (linear) moment-to-moment influence between the spouses, since the 95 % credible intervals for the average (level-2) influence in both directions included zero. To check whether this was caused by some couples having an opposite influence pattern of other couples, we also inspected the 95 % credible intervals for the individual couples, and these included zero for 89.9 % of the husband-to-wife influence parameters, and 93.8 % of the wife-to-husband influence parameters. Thus, we concluded that there was little evidence of linear influence between the spouses at this time lag. Note that this result is in line with the previous findings of Madhyastha et al. (2011), who analyzed the data of each couple separately using single-case TAR and AR models with diverse influence functions.

**Fig. 4:**
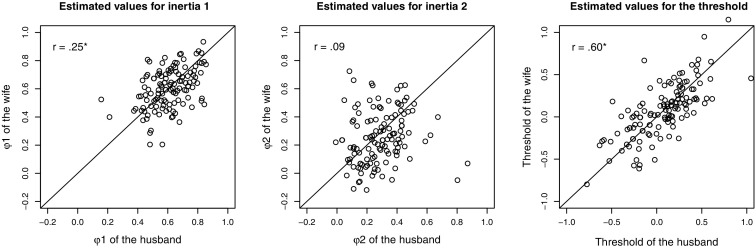
*Scatterplot* of the estimated level-1 inertias and thresholds, comparing husbands with their wives. Each *point* represents a *couple*. *Points* on the *diagonal (equality) line* represent couples where the husband and wife had the same inertia/threshold, and $r$ is the model estimate for the correlation between the parameters of husbands and wives (*asterisk* indicates that zero was not contained in the 95 % credible interval of the correlation).

Lastly, to investigate whether couples resembled each other in their regulation of affective behavior, we inspected the correlations between the inertias and threshold for the husbands, and those for the wives. The individual estimates for the husbands and wives are depicted in the scatterplots in Figure 4 together with the correlations. The estimated correlation between husbands’ and wives’ inertia in the lower state was $r_{\phi _1 }= .25$, with a 95 % credible interval of $[0.05, 0.45]$, indicating that spouses tended to resemble each other in their regulation of negative affective behavior. However, the estimated correlation between husbands’ and wives’ inertia in the upper state $r_{\phi _2}$ was only $.09$, with a 95 % credible interval of $[-0.15, 0.33]$, so that we could not conclude that there was similarity between spouses in their regulation of positive affective behavior. Lastly, the estimated correlation between the husbands’ and wives’ threshold was $r_{\tau } = .60$, with a 95 % credible interval of $[0.46, 0.73]$, indicating quite high similarity between the spouses in their equilibrium of affective behavior.

In summary, we established that the majority of both the husbands and the wives had state-dependent regulation of affective behavior, and that negative behavior was more persistent than positive behavior. Furthermore, the results indicated that husbands and wives were more similar to each other than to others in the sample, when considering their regulation of negative affective behavior, but no such relationship was found with respect to the regulation of their positive affective behavior. They were, however, very much alike with respect to their affective equilibrium. Note further that we did not find any evidence for gender differences in the regulation of affective behavior. In conclusion, these findings illustrate the usefulness of the new multilevel TAR model, as well as the importance of considering the dependence between spouses’ characteristics in dyadic research.

### Application 2: Daily Fluctuations in Negative Affect

**Fig. 5:**
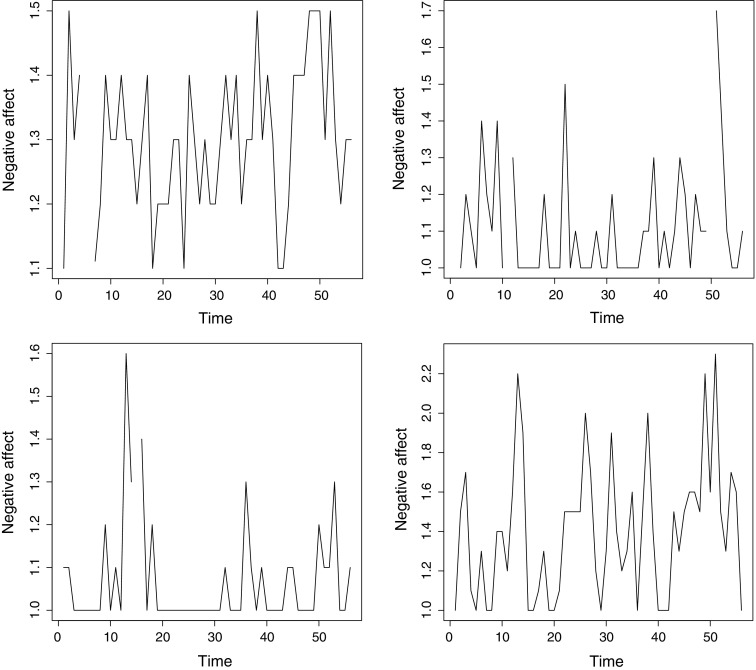
Negative affect scores of four example subjects over the course of the 56 study days.

For an additional empirical illustration, we sought a data set including a person-level predictor variable, so that we could model the inertias and threshold. We analyzed a data set including a measure of trait neuroticism in addition to daily self-report measures of negative affect. These data were obtained from the older cohort (ages 50 and higher, $N=304$) of the Notre Dame Study of Health & Well-Being. Specifically, we considered the Negative Affect subscale of the PANAS (Watson, Clark, & Tellegen, 1988), which the participants filled out on 56 consecutive days. These daily scores ranged from 1 (very little or no negative affect) to 5 (very intense negative affect) in 0.1 increments, but as noted before by Wang, Hamaker, and Bergeman (2012), the negative affect scores of many individuals showed little variation over the course of the study. Some persons’ scores showed a floor effect because they repeatedly reported experiencing no negative affect whatsoever (score 1). Although skewed data can be generated by a TAR process with a large inertia difference, the lack of variation in scores violates the model’s assumption of normally distributed residuals (innovation). Thus, we chose to apply the multilevel TAR model only to those individuals whose negative affect scores had a standard deviation of 0.1 or higher. This criterion excluded the most stable score patterns, while avoiding overly subjective decisions on which participants to include. Figure 5 portrays the affect scores of four of the included individuals.

As an additional criterion, we only included the persons who had no more than six missing scores on the negative affect variable, so that at least fifty scores per person were available. Because the data also contained scores for trait neuroticism (using the NEO PI-R; Costa & McCrae, 1992), we could investigate the relationship between neuroticism and affect regulation. Of the 224 persons with sufficient data, 161 persons (71.9 %) met our criterion of sufficient variance in the negative affect scores and were used in the analysis. Although 96 of these persons (59.6 %) had between one and six missing observations this poses no problem for the analysis, since OpenBUGS automatically implements Bayesian multiple imputation for dependent variables.

#### Hypotheses

We investigated whether or not the daily regulation of negative affect was, on average, state-dependent, by estimating the multilevel TAR model and inspecting the 95 % credible interval of the mean difference in inertia between the two states of more intense and less intense negative affect. We also investigated how neuroticism related to the dynamics of affect regulation. As mentioned before, Suls et al. (1998) found a positive relationship between neuroticism and the general inertia parameter $\phi$ of the AR model, but now we hypothesized an underlying pattern where more neurotic individuals have weaker affect regulation only or especially for increased negative affect. In other words, we proposed that the TAR model may be more appropriate than the AR model and we expected to see a more positive difference $\phi _2 - \phi _1$ for more neurotic individuals, compared to less neurotic individuals. To address this hypothesis most directly, we made use of a model parameterization with $\phi _1$ and $\delta$, where $\delta$ equals the difference $\phi _2 - \phi _1$, so that $\phi _2$ was implicitly defined. We also expected that neurotic individuals have a higher equilibrium (i.e., threshold) of negative affect than less neurotic individuals. In order to test both hypotheses, we included neuroticism as a (centered) level-2 predictor of the random inertia, inertia difference, and threshold.

Writing $x_i$ for person $i$’s centered neuroticism score, the model equations are:

$$\begin{aligned} \begin{array}{l} Y_{t,i} = \left\{ \begin{array}{rl} \tau _i + \phi _{1,i} (Y_{(t-1),i} - \tau _i) + \epsilon _{t,i} &{}\qquad \text {if } \qquad Y_{(t-1),i} < \tau _i\\ \tau _i + \phi _{2,i} (Y_{(t-1),i} - \tau _i) + \epsilon _{t,i} &{} \qquad \text {if }\qquad Y_{(t-1),i} \ge \tau _i \\ \end{array} \right\} , \\ \begin{array}{ccl} \epsilon _{t,i} &{} \sim &{} \mathcal {N} (0,\sigma ^2_{\epsilon }), \\ \phi _{1,i} &{} = &{} \gamma _{\phi _1} + \beta _1 x_i + u_{\phi _1,i} , \\ \phi _{2,i} &{} = &{} \phi _{1,i} + \delta _{i} \\ \delta _i &{} = &{} \gamma _{\delta } + \beta _2 x_i + u_{\delta ,i}\\ \tau _{i} &{} = &{} \gamma _{\tau } + \beta _3 x_i + u_{\tau ,i}, \\ \begin{bmatrix} u_{\phi _1,i} \\ u_{\delta ,i} \\ u_{\tau ,i} \\ \end{bmatrix} &{} \sim &{} \mathcal {N} \left( \begin{bmatrix} 0 \\ 0 \\ 0 \end{bmatrix} , \begin{bmatrix} \sigma ^2_{\phi _1} &{} \sigma _{\phi _1 \phi _2} &{} \sigma _{\phi _1 \tau } \\ .&{} \sigma ^2_{\phi _2} &{} \sigma _{\phi _2 \tau } \\ .&{}. &{} \sigma ^2_{\tau } \end{bmatrix} \right) , \end{array} \end{array} \end{aligned}$$

where the fixed effects $\gamma _{\phi _1}$ and $\gamma _{\tau }$ represent the predicted lower state inertia and threshold for a person with average neuroticism. The predicted upper state inertia for a person with average neuroticism is indirectly given by $\gamma _{\phi _1} + \gamma _{\delta }$. The BUGS syntax used for estimating this model is included in Appendix 3.

#### Results

We ran the model with 2 parallel MCMC chains using different starting values. We used 10,000 burnin iterations, after which the convergence was adequate and 50,000 samples (per chain) were used for inference. This analysis took 79 min on a system with 16 GB RAM, running on a single core with 3.40 GHz processing speed.

**Fig. 6:**
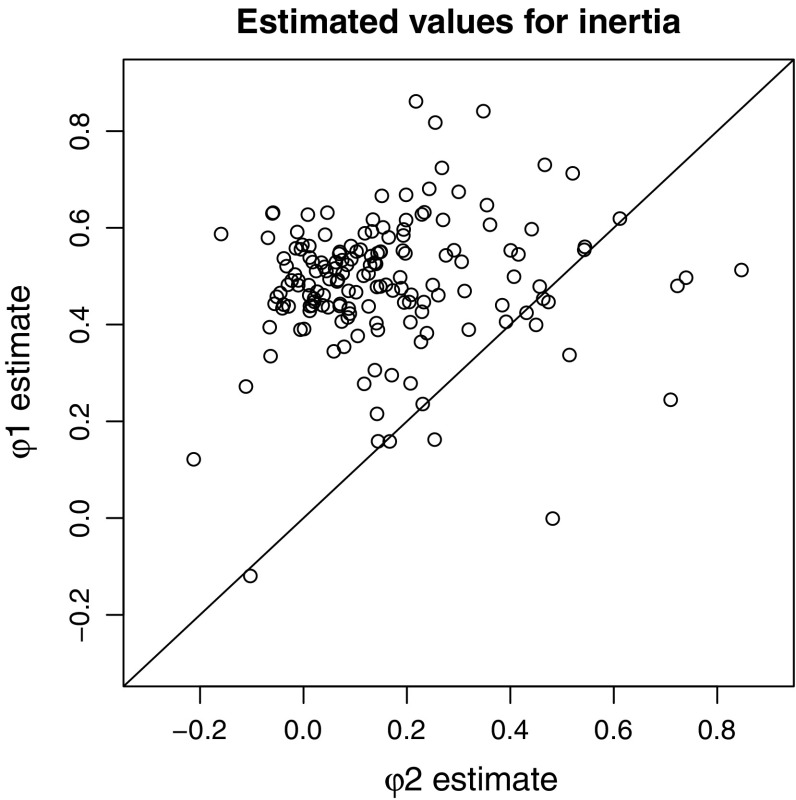
Scatterplot of the estimated level-1 inertias for less intense negative affect ($\phi _1$) and more intense negative affect ($\phi _2$). Since most of the points fall above the *diagonal line*, we can conclude that the majority of the persons was characterized by stronger regulation during episodes of more intense negative affective behavior. The implication is that they experienced prolonged episodes with only little negative affect, and when they did experience more intense negative affect, they recovered quickly.

Based on the estimates for the level-2 inertia difference ($$\gamma _{\delta } = -0.33$$, with a 95 % CI of $$[-.43, -.22] $$) we conclude that the regulation of negative affect depended on affect intensity. The estimates for the mean inertias ($$\gamma _{\phi _1} = .49, \gamma _{\phi _2} = .16$$) indicate that the average person had a lower inertia during episodes of more intense negative affect (with effect size Cohen’s $$d = 2.4$$). The inertia estimates for the individual persons are depicted in Figure 6. Considering the threshold ($$\gamma _\tau = 1.33$$), these results together reflect the fact that, even in the subsample that we selected for analysis, many of the persons reported experiencing very little negative affect on most days. When they did experience more intense negative affect, they recovered quickly. We see that even though these data do not perfectly meet all the assumptions of the TAR model, the estimated model parameters do provide meaningful information. The relatively high mean inertia parameter for the lower state reflects the stability by which the average person could maintain an absence of negative affect for prolonged periods, while the low inertia parameter for the upper state indicates that the average person was very quick to recover from an episode of increased negative affect.

The effect of neuroticism on the equilibrium (i.e., on the threshold $\tau$) was $\beta _3 = 0.02$, with a 95 % credible interval of $[0.01, 0.03]$, lending support to our hypothesis that more neurotic individuals have an equilibrium that reflects higher levels of negative affect. The coefficient indicates that a 10-point increase in trait neuroticism predicts a 0.2-point increase in the equilibrium of negative affect (measured on a 5-point scale). Since the neuroticism scores in this sample varied between 14 and 43, with a standard deviation of 5.4, this can be considered a small effect. Contrary to our expectation, we did not find a distinguishable effect (based on the 95 % CI) of neuroticism on the inertia difference $\delta$, nor was there any distinguishable relationship between neuroticism and the inertia for episodes of less intense negative affect. A possible explanation for these null findings may be that the variability in inertia and in inertia difference in a multilevel TAR model may be smaller than the variability in inertia in a multilevel AR model. Also, since there are fewer data points per inertia parameter, the power to detect relationships between the inertias and other variables may be lower than in an AR model.

In conclusion, we found no evidence for a relationship between trait neuroticism and the regulation of negative affect, for the persons in our subsample. Our analysis did indicate that more neurotic individuals tended to experience slightly more intense negative affect. We refrain from generalizations to a broad population, since we analyzed only a subset of the data, and many of the elderly persons in this sample reported constant low levels of negative affect throughout the study. Since there are indications that elderly people experience less negative affect (Carstensen, Fung, & Charles, 2003; Charles, Reynolds, & Gatz, 2001), we expect that studies with younger participants will find more fluctuations in negative affect. It may also be of special interest to study clinical subpopulations known to have different affective experiences than the average population. In conclusion, we note that the TAR model was clearly preferable to an AR model for these data, since affect regulation was state-dependent for most of the individuals in the sample and a multilevel AR model would misrepresent the underlying regulatory process. This illustrates the importance of differentiating between low-intensity and high-intensity affect when studying inertia.

### Conclusions

The results of the two empirical applications only partly matched our specific substantive expectations. However, in both data sets we did find evidence that the strength of regulation varies considerably within persons, depending on the intensity of their affect, and this underlines the relevance of the proposed modeling approach. The two applications illustrate that the basic multilevel TAR model can be modified or extended to address different research questions. Importantly, these examples also demonstrate that the application of the modeling approach is not limited to a specific research design, since the two empirical data sets were different in multiple regards. The first application was based on observational data obtained from dyads to study their moment-to-moment behavior on an affective dimension running from negative affect to positive affect. In contrast, the second application concerned daily fluctuations in the intensity of experienced negative affect, based on daily self-reports by individuals.

## General Discussion

We presented a multilevel TAR model for studying within-persons and between-persons differences in regulatory strength, and applied this model to the study of affect regulation. Previous studies have concluded that weak affect regulation, as a general trait, is related to neuroticism, depression and low self-esteem (Kuppens et al., 2010; Suls et al., 1998; Wang et al., 2012). The multilevel TAR model makes it possible to investigate whether these relationships hold for affect regulation in general, or only/especially for affect regulation during episodes that are, for instance, characterized by intense negative affect. The two empirical applications in this paper demonstrate the potential of the proposed modeling approach.

Based on the results of our simulations, we conclude that Bayesian estimation of the multilevel TAR model is feasible and that it leads to appropriate inferences regarding the fixed effects. These good results were obtained despite using the suboptimal inverse Wishart prior for covariance matrices, so we may expect even better performance when more uninformative priors are developed. Furthermore, we demonstrated an effective approach for distinguishing between a multilevel AR model and a multilevel TAR model. With regard to the sample size required for valid application of this modeling approach, we are hesitant to speak of sufficient sample sizes, since a large number of observations with little score variation over time (as in our second empirical application) does not necessarily provide richer information than a smaller sample with more fluctuations. Given the complexity of the model, which includes autoregression coefficients for two AR processes for of each person, researchers should aim to obtain at least 50 measurements per person even when the expected effect sizes are large.

The modeling approach that we presented is intended as a basic framework, that can be used whenever researchers expect to find a regulatory mechanism operating over time, and want to investigate whether this regulation is dependent on a threshold variable. While the current study focused on affect regulation, the model may also be useful for studying cognitive, behavioral, or physiological processes. Besides self-report measurements and observational data, the model can also be applied to experimental data or physiological measurements, both of which frequently involve many observations per person.

In addition to the model extensions presented in the empirical applications, here we list some further options for adapting the basic multilevel TAR model to various research questions. One option is to allow the effect of time-varying (level-1) predictors to be state-dependent as well. For example, an affect researcher could investigate whether the impact of an emotionally charged event depends on the state the person is currently in. Alternatively, the impact of such an event can be allowed to depend on observed level-2 variables such as personality traits, in line with the findings of Wichers et al. (2007). In a different vein, if the data set is sufficiently large, one can also consider multilevel TAR models with more than two states, reflecting different regulatory mechanisms. Finally, we note that it is also possible to use a different time-varying threshold variable, as long as this threshold variable is observed.

Application of the modeling approach presented here presumes that the interest is in discrete states, based on an observed threshold variable, that underlie the regulatory process. Researchers interested in models where regulation depends on an observed *continuous* process may consider as an alternative the multilevel smooth transition autoregressive (STAR) model (Fok, Van Dijk & Franses, 2005). If it is assumed that there are discrete states underlying the data, but there is no available threshold variable, one can consider Markov switching models such as the Markov switching AR model (Hamilton, 1989; Frühwirth-Schnatter, 2006).

The multilevel TAR model has some caveats that are important to mention at this point. First, the residuals (innovations) in the model are assumed to be normally distributed, which calls for continuous data with a sufficient level of within-person variation. Further simulations are needed to determine the robustness of the model estimates when this assumption is violated, for example, when the data are distributed more like in our second empirical application. A second caveat of the model is that the observed scores for each person are treated as a stationary process, meaning that there should be no time trend in their data. Lastly, an important assumption underlying the AR and TAR models is that the intervals between consecutive measurements are (approximately) equal. This last assumption clearly does not hold for data collected with the experience sampling method (ESM), involving measurements at varying intervals. Such data may be analyzed using the hierarchical Ornstein–Uhlenbeck (OU) model proposed by Oravecz, Tuerlinckx, and Vandekerckhove (2009), which is the continuous time extension of the multilevel AR model. However, there is no continuous time multilevel TAR model available yet. When the multilevel TAR model presented in this paper is applied to data with varying time intervals, theoretically, this will add noise to the inertia estimates and decrease the power of the analysis. That said, it is not clear whether this is problematic in practice: The multilevel AR model is based on the same assumption but it has been applied succesfully to ESM data to uncover affect regulation dynamics (e.g., Koval & Kuppens, 2011).

In sum, given the results from our simulation study and the empirical applications, we conclude that the multilevel TAR model is a valuable addition to the available techniques for analyzing intensive longitudinal data. Although there are some caveats, this modeling approach opens up new possibilities for in-depth study of regulatory processes operating at diverse time scales.

### Electronic supplementary material

Below is the link to the electronic supplementary material.

Supplementary material 1 (txt 260 KB)

Supplementary material 2 (txt 0 KB)

## Appendix 1: BUGS-Syntax for the Basic Multilevel AR and TAR Models

The syntax files below are used to fit the multilevel AR model from Eq. 2 and the multilevel TAR model from Eq. 4. Sometimes the notation diverges from these equations, because in BUGS we write out the distribution for the random effects directly, as in $\phi _{i} \sim \mathcal {N}(\gamma _{\phi }, 1/\sigma ^2_{\phi })$, rather than using the deviation terms such as $u_{\phi ,i}$. Note that the normal density in BUGS has a mean and *inverse* variance.

The data input should be a matrix $Y$, with rows for observations, and columns for persons, containing uncentered (or only grand-centered) scores and *NA* for missing values. Missing scores on the first measurement occasion are not allowed, but see Appendix 3 for a solution. Our syntax specifies vague priors for all model parameters, but different, more informative priors can also be used if desired.

## Appendix 2: BUGS-Syntax for the Bivariate TAR Model with Mutual Linear Influence at Lag 1

The data input is two matrices named ***H*** and ***W***, each having rows for observations, and columns for persons, containing the uncentered (or only grand-centered) scores and *NA* for missing values. Missing scores on the first measurement occasion are not allowed, but see Appendix 3 for a solution.

## Appendix 3: BUGS-Syntax for the TAR Model with a Level-2 Predictor

The data input is a matrix named $Y$, with rows for observations, and columns for persons, containing the uncentered scores and *NA* for missing values. In addition, a vector $x$ with the scores on the (person-level) predictor neuroticism is provided. These scores don’t have to be centered, for BUGS will center them as part of the model estimation. Another vector ***firstT*** gives the first time point at which each person was observed, to accommodate missingness on the first measurement.

## Footnotes

1 Results not included here, but obtainable from the first author upon request.

2 Although 100 replications is a relatively small number in simulation research, the estimation in OpenBUGS was rather time-consuming and since the pattern of results was clear enough, we saw no need for increasing the number of replications.

3 Results not included here, but obtainable from the first author upon request.
